# Advanced aging effects on implicit motor imagery and its links to motor performance: An investigation via mental rotation of letters, hands, and feet

**DOI:** 10.3389/fnagi.2022.1025667

**Published:** 2022-11-17

**Authors:** Hiroyuki Muto, Maki Suzuki, Kaoru Sekiyama

**Affiliations:** ^1^Institute for the Future of Human Society, Kyoto University, Kyoto, Japan; ^2^Division of Cognitive Psychology, Faculty of Letters, Kumamoto University, Kumamoto, Japan; ^3^Department of Behavioral Neurology and Neuropsychiatry, Osaka University United Graduate School of Child Development, Suita, Japan; ^4^Graduate School of Advanced Integrated Studies in Human Survivability, Kyoto University, Kyoto, Japan

**Keywords:** motor imagery, mental rotation, advanced aging, cognitive aging, embodied cognition

## Abstract

This study focuses on changes in implicit motor imagery during advanced aging and these changes’ co-occurrences with physical motor deficits. We administered a mental rotation (MR) task with letters, hands, and feet to 28 young adults (20–27 years) and to 71 older adults (60–87 years), and assessed motor skills (gait mobility and hand dexterity) and neuropsychological performance. Compared to young adults, older adults showed lower MR performance for all stimuli and stronger biomechanical constraint effects on both hand and foot rotation. Moreover, the foot biomechanical constraint effect continued to increase during late adulthood, and declines in hand and foot motor imagery emerged at earlier old ages than declines in visual imagery. These results first demonstrated distinct aging trajectories of hand motor imagery, foot motor imagery, and visual imagery. Exploratory partial correlation analysis for older adults showed positive associations of low-level perceptual-motor skills (Trail Making Test-A performance) with hand and foot MR performance and positive associations of mobility (Timed Up and Go test performance) with foot and letter MR performance. These associations exhibited somewhat different patterns from those of young adults and raised the possibility that age-related declines in motor (and visual) imagery co-occur with declines in motor functioning.

## Introduction

According to the theoretical framework of embodied cognition, human cognitive processes are grounded in bodily interactions with the physical world (Wilson, 2002; Waller, 2014). Consistent with this framework, much research has demonstrated sensorimotor processes’ role in a wide range of cognitive functions (e.g., Glenberg and Kaschak, 2002; Dijkstra et al., 2007; Muto et al., 2018; Nagai et al., 2019). In recent years, increasing attention has been paid to incorporating and validating the embodied cognition framework in aging research (for reviews, see Vallet, 2015; Costello and Bloesch, 2017; Kuehn et al., 2018). This attention seems a natural consequence of physical and cognitive declines’ tendency to occur concurrently in normal aging (for a review, see Roberts and Allen, 2016), and this concurrence has potential impacts on clinical applications (Camicioli et al., 1998; Verghese et al., 2002; Waite et al., 2005; Sakamoto et al., 2007). Thus, elucidating links between older adults’ physical and cognitive functioning is theoretically and clinically important. Aligned with this framework, the current study focuses on age-related changes in motor imagery during healthy advanced aging and on those changes’ associations with physical motor performance.

### Implicit and explicit motor imagery

Motor imagery means the mental simulation of an action without overt physical action, evoked explicitly or implicitly (McAvinue and Robertson, 2008). While explicit motor imagery is investigated through tasks in which participants are instructed to imagine actions such as reaching for a visual target (e.g., Sirigu et al., 1996) and hand gripping (e.g., Osuagwu and Vuckovic, 2014), implicit motor imagery is studied through tasks without direct instructions (detailed below). Motor imagery, regardless of explicit or implicit evocation, is tightly linked to motor planning and execution, as evidenced by myriad studies revealing neural overlap (Parsons et al., 1995; Kosslyn et al., 1998; Sekiyama et al., 2000; Vingerhoets et al., 2002; de Lange et al., 2006; Osuagwu and Vuckovic, 2014).

The most typical experimental task in studying implicit motor imagery is mental rotation (MR) of hands (or the “hand laterality” task) in which participants see a picture of a hand in various orientations and identify whether it is the left or right (Cooper and Shepard, 1975). A number of studies have demonstrated that participants’ reaction time (RT) lengthened as the angular disparity between the hand’s presented and canonical orientations became larger, suggesting that participants mentally rotated their own hands. Besides this angle effect, rotational direction influences RT as well (Sekiyama, 1982; Parsons, 1987); participants’ response to laterally rotated hands (fingers pointing away from the body’s midline) took longer than to medially rotated hands (fingers pointing toward the body’s midline). This medial–lateral effect seems to reflect the joints’ biomechanical constraints. Indeed, RT patterns mirror actual hand movements’ rated physical difficulty (Sekiyama, 1983) and temporal properties (Parsons, 1994), supporting correspondence between physical and mental movements. Additionally, the MR of feet (or the “foot laterality” task) shows both the angle effect and the medial–lateral effect as reflecting biomechanical constraints imposed by the lower limbs’ joints (Parsons, 1987; Ionta et al., 2007), although this has been less studied. We focus on implicit motor imagery, which can be measured by the MR task more objectively, as opposed to explicit motor imagery.

### Aging effects on implicit motor imagery

Several previous studies have investigated age-related declines in hands’ MR, with mixed results. Saimpont et al. (2009) found that older adults (mean age: 78.3 years; range: 75–87 years) exhibited longer RTs than young adults, especially for hands in anatomically awkward orientations. De Simone et al. (2013) obtained similar results when comparing older adults (mean age: 71.9 years; range unreported) with young adults. A study using a paper-and-pencil test (Iachini et al., 2019) showed the same conceptual results: Older adults (mean age: 67.2 years; range: 60–82 years) responded less accurately to hand MR items than young adults. However, an fMRI study by Zapparoli et al. (2016) failed to detect RT differences between older (mean age: 61 years; range unreported) and young adults.

As Zapparoli et al. (2016) discussed, this discrepancy might be attributed to at least two causes. First, older participants’ mean age was younger in Zapparoli et al. (61 years) than in the other studies (67, 72, and 78 years). Implicit motor imagery deficits possibly become salient during advanced aging, especially because explicit motor imagery adheres to Fitts’s law in both young adults aged 19–25 years and older adults aged 62–67 years but not in older adults aged 71–75 years (Skoura et al., 2005). Second, while Zapparoli et al. analyzed RTs of hand MR subtracted by choice RTs for each subject (ΔRTs) to control older adults’ generally slowing motor responses, the other studies did not. Such a control is needed for precise investigations of motor imagery declines’ distinct features over the lifespan. These considerations lead to the necessity of examining potential advanced aging effects on implicit motor imagery when controlling for the general slowing.

Whether findings on the aging of implicit motor imagery are specific to hands or can be extended to other body parts remains unclear; this is because, to the best of our knowledge, no studies have considered the aging process through the MR of feet. Also, older adults’ common motor deficits are known to involve gait control (Ketcham and Stelmach, 2004), and the tactile acuity of the feet (and hands) is more vulnerable to aging than that of other body regions (Stevens and Choo, 1996; Stevens et al., 2003). As described by the embodied cognition framework, such a physical weakening in the lower limbs during aging could lead to a decline in implicit motor imagery for the lower limbs. Thus, the present study employs an MR task of both hands and feet.

### Contrast between motor and visual imagery

Besides motor imagery, aging also impairs visual imagery, that is, the construction, maintenance, and transformation of internal visual representation. A typical task for assessing visual imagery’s transformational process is the MR of letters, in which participants judge whether a rotated alphanumeric character is normal or mirrored (Cooper and Shepard, 1973). This task’s RT increases with angle but is rarely affected by rotational direction, unlike the MR of body parts. Using various tasks, previous literature has confirmed age-related declines in mental visual transformations (Cerella et al., 1981; Berg et al., 1982; Dror and Kosslyn, 1994; Gondo et al., 1998; Briggs et al., 1999; Band and Kok, 2000; De Simone et al., 2013; Iachini et al., 2019; Zhao et al., 2019; Muto et al., 2020). Theoretically, mental visual transformations are classified as object-based because they involve transformations of external objects in the environment. Conversely, transformations of body parts are classified as egocentric because they entail referring to one’s own body axes (for a review, see Zacks and Michelon, 2005).

Some studies (De Simone et al., 2013; Kaltner and Jansen, 2016) have suggested that aging affects egocentric transformations (related to motor imagery) more than object-based transformations (related to visual imagery). For example, in De Simone et al. (2013), while older adults exhibited worse performance than young adults on an egocentric transformation task (hand MR), the two groups showed no difference in an object-based transformation task (location judgment of a marker placed on a hand). Interestingly, Zapparoli et al. (2016) reported that older adults had greater neural activation in occipital regions than young adults; this difference was greater for the MR of hands than of letters, although behavioral performance showed no group difference. This finding was interpreted as evidence that older adults use relatively intact visual imagery to compensate for impaired motor imagery. To contrast aging trajectories of motor and visual imagery, the present study includes the MR of letters along with the MR of hands and feet. From the above discussion, one may predict that the performance difference from young adults emerges at a relatively earlier age for motor imagery than for visual imagery.

### Links between physical and cognitive declines

The key assumption of the embodied cognition framework is interdependence between physical and cognitive functioning, which predicts impairments’ co-occurrence during the aging process. Assessing hand dexterity and gait mobility is useful for testing this prediction because the two can be easily measured and relate to cognitive functions in both healthy and clinical populations of older adults. In mobility, for example, gait dysfunction can predict subsequent cognitive impairment and dementia (Camicioli et al., 1998; Verghese et al., 2002; Waite et al., 2005), and healthy older adults’ mobility, measured by the Timed Up and Go test (TUG; Podsiadlo and Richardson, 1991), is associated with their executive function (Herman et al., 2011; Donoghue et al., 2012), visually encoded working memory (Kawagoe and Sekiyama, 2014; Kawagoe et al., 2015), and accuracy for object-based MR of whole bodies (Jansen and Kaltner, 2014). Also, associations between dexterity, measured by a pegboard test, and general cognitive functions were reported in studies on older adults with mild cognitive impairment (MCI) and dementia (Kluger et al., 1997; Sakamoto et al., 2007) as well as those on healthy older adults (Yoon et al., 2010). Thus, we assess dexterity and mobility as motor performance indices. Although these tests were not intended to measure young adults’ motor functions, it may be important to conduct a comparative analysis with young adults’ data to determine whether the observed associations are unique to older adults. Additionally, we administered neuropsychological tests for exploratory purposes of examining associations with motor and visual imagery.

### Present study

In brief, through the lends of the embodied cognition framework, the present study investigated age-related changes in implicit motor imagery during advanced aging and these changes’ co-occurrence with physical motor deficits. The novelty of the present study can be summarized as follows. First, to examine advanced aging effects, we sampled a sufficient number of older adults in their 60, 70, and 80 s as well as young adults in their 20 s. Second, in addition to the hand MR, we administered the foot and letter MR to extend findings on the hand MR to lower limb motor imagery and to contrast trajectories of declines in motor and visual imagery. Third, we explored how MR performance relates to motor and neuropsychological performance.

Specifically, the present study’s four purposes are as follows: (1) confirm that the older group exhibits worse performance on average than the young group for all MR types; (2) test whether performance on each MR type continues declining even during advanced aging; (3) inspect whether performance differences from the young group become pronounced at earlier old ages for the hand and foot MR than for the letter MR (i.e., motor imagery starts to decline earlier than visual imagery); and (4) explore which indices of motor and neuropsychological performance relate to each of the three MR types in older adults when controlling for age, gender, and education years. Further, this explores whether these relations are distinct from those of the young group.

## Materials and methods

### Participants

Participant inclusion criteria were (1) age (the 20, 60, 70, or 80 s), (2) normal or corrected-to-normal vision, (3) right-handed, (4) no medical history of neurological disorders, (5) no psychoactive drugs, sleeping pill, or tranquilizer taken, and (6) no marked difficulty in moving the limbs or walking. Based on the sample sizes of similar previous studies (Saimpont et al., 2009; De Simone et al., 2013; Zapparoli et al., 2016), we planned to collect data from 20 or more participants for each age group (20, 60, 70, and 80 s) within limits of available participants and research resources. Thus, we recruited 31 young and 91 older adults from Kumamoto University and surrounding communities. This study was approved by the Ethical Committee of Kumamoto University (no approval number; approval date: July 8, 2014). All participants gave written informed consent before participating.

We excluded data from the following: (1) 11 older participants who scored below 26 on the Japanese version of the Mini-mental State Examination (MMSE; Folstein et al., 1975); (2) one older participant who scored lower than the means minus 1.5 standard deviations (*SD*s) for the Japanese version of the Wechsler Memory Scale-Revised (WMS-R) Logical Memory II (Wechsler, 1987; Kawano, 2012); (3) one young and six older participants with medical history of neurological disorders; (4) one older and two young participants who took psychoactive drugs; and (5) one older participant whose experimental data were not recorded due to computer errors. Thus, we analyzed remaining data from 28 young adults (8 men, 20 women; mean age = 22.2 years, range 20–27 years) and 71 older adults (16 men, 55 women; mean age = 73.5 years, range 60–87 years). Mean years of education were 15.8 (*SD* = 0.8) for the young group and 12.6 (*SD* = 2.3) for the older group, whose mean age did not significantly differ between men (72.8 years) and women (73.7 years), Welch’s *t*(23.3) = 0.425, *p* = 0.675. We confirmed that all participants were right-handed according to the Edinburgh Handedness Inventory (Oldfield, 1971).

### Motor assessment

To assess hand dexterity, we conducted the pegboard test as in our previous work (Kawagoe and Sekiyama, 2014). We used a wooden pegboard (Sakai Medical Corporation SOT-2102) with 20 pegs (1.4 cm in diameter, 5.0 cm long) and a board (28.0 cm wide, 23.0 cm deep, and 2.2 cm high) with four parallel rows in a total of 20 holes. All 20 pegs were placed into the holes at the beginning. We placed the pegboard in front of the midline of the participant’s body. We ensured that the test setup was equal among participants. Participants were verbally instructed to use their right hand to turn all 20 pegs upside down; performance time was measured using an electronic stopwatch, which started at the experimenter’s “start” signal and stopped when the participant finished turning the final peg upside down. We provided the same instructions to participants in both age groups based on a script. All participants were tested twice; they first performed the pegboard test at normal speed to familiarize them with the task and then as quickly as possible. We included only the second measurement in our analysis. Almost all participants performed faster in the second test than in the first test (27 of 28 young adults and 65 of 71 older adults); hence, they could be considered to have correctly understood the instruction. The other participants spent more time performing the second trial compared with the first trial, but the time difference was below 2 s.

Mobility was assessed with TUG (Podsiadlo and Richardson, 1991). Participants were verbally instructed to stand from an armless chair, walk 3 m toward a marker, make a turn around a cone placement, walk back to the chair, and sit down. We provided the same instruction to all participants according to the standardized procedure and carefully set the test environment to be equal among them. We measured performance time using an electronic stopwatch, which started when the participant’s buttocks left the chair and stopped when the participant’s buttocks touched it. All participants wore athletic/walking shoes. Performance time was recorded in two trials, first at normal speed and then as fast as possible. Again, we included only the second measurement in analysis. All participants performed the second trial faster than the first, suggesting that they correctly understood the instruction.

### Neuropsychological assessment

All participants completed a battery of standardized neuropsychological tests: the MMSE, WMS-R Logical Memory (LM) I and II, the WMS-R Digit Span Test (DST), the WMS-R Spatial Span Test (SST), the Trail Making Test (TMT), and the copy condition of the Rey–Osterrieth complex figure test (ROCFT). The MMSE (with a cut-off score of 26) and LM II were used to screen for cognitive impairments. Since the Japanese version of the WMS-R manual (Sugishita, 2001) did not provide standardized scores for examinees over 75 years, we calculated cut-off scores (i.e., the mean minus 1.5 *SD*s) using Kawano’s (2012) age-appropriate statistics.

The Japanese version of the TMT (Kashima et al., 1986; Reitan, 1992), composed of two parts, measured visual attention and executive function. On the TMT-A, participants connected randomly arranged numbers from 1 to 25 in ascending order (i.e., 1-2–3-4…). Time to complete the TMT-A reflects relatively low-level perceptual processing, for example, visual scanning and motor speed. On the TMT-B, participants alternately connected numbers (1–13) and hiragana (a Japanese alphabet; あ to し) in numeric and alphabetical order (i.e., 1-あ-2-い…). Time to complete the TMT-B reflects executive function (i.e., task switching) in addition to low-level perceptual processing. Furthermore, we calculated ΔTMT (TMT-B time minus TMT-A time) as a purer measure of task switching and executive function (Corrigan and Hinkeldey, 1987; Drane et al., 2002).

The ROCFT (Meyers and Meyers, 1995) assessed visuoconstructional ability. Participants copied, as accurately as possible, a complex figure printed on paper. Based on the test’s original scoring criteria, their performance was scored from 0 to 36.

The DST and SST, subtests of the WMS-R (Wechsler, 1987), measured short-term memory and working memory capacities. All participants completed both forward and backward versions of the DST and SST. Based on original criteria, scores ran from 0 to 14 for the forward SST and from 0 to 12 for the others.

### Experimental stimuli

**Figure 1:**
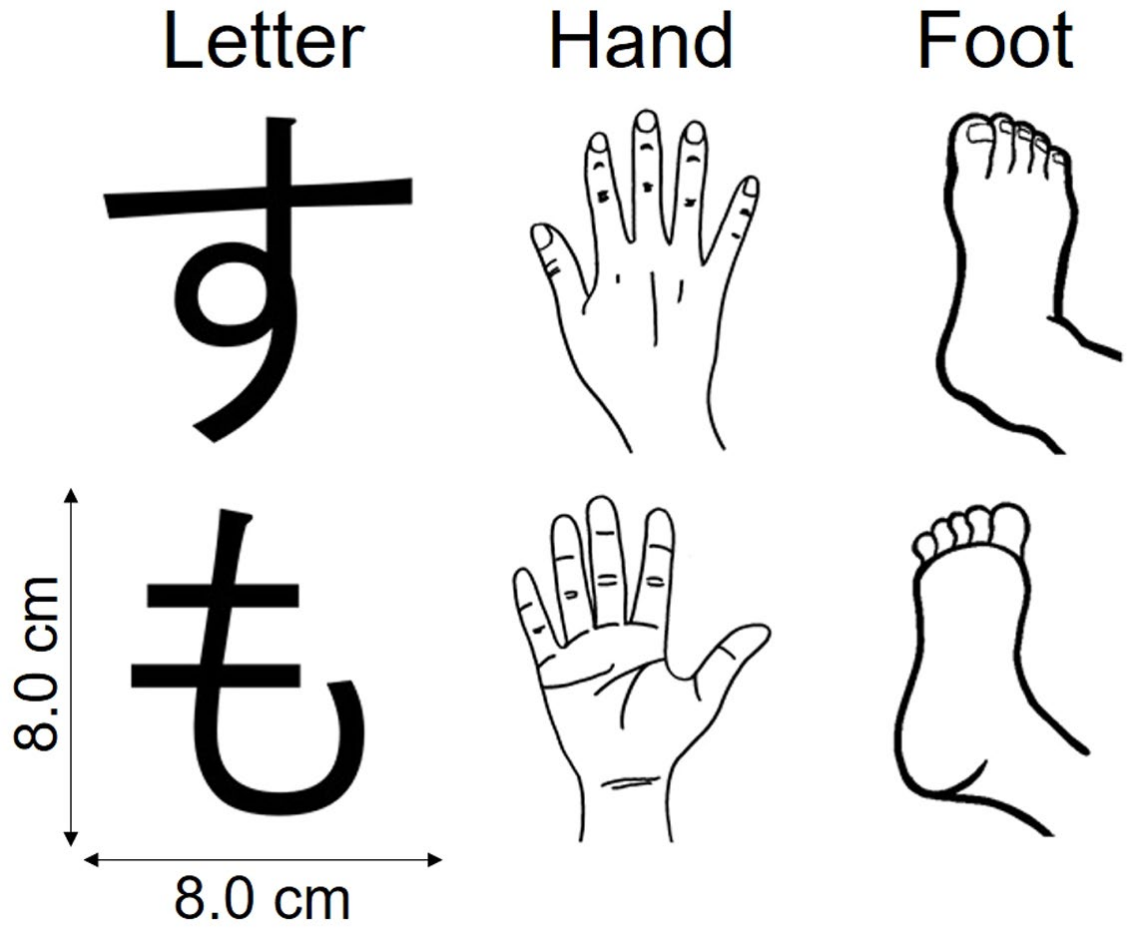
Stimuli used in the mental rotation task. These pictures and their mirrored counterparts were presented at 45°, 90°, and 135°, clockwise and counterclockwise.

For the MR task, we used three types of stimuli—letters, hands, and feet (Figure 1)—to measure visual imagery, hand motor imagery, and foot motor imagery, respectively. Letter stimuli were normal and mirrored images of Japanese kana letters “す” and “も.” Hand stimuli were line drawings of left and right hands, each with the back or palm view. Foot stimuli were line drawings of left and right feet, each with the top or sole view. Each stimulus was presented in any of six orientations in the picture plane: 45°, 90°, and 135°, clockwise and counterclockwise. For the choice-RT task, we used Arabic numerals “1” and “2.” All stimuli appeared against a white background, centered on a 15.4-in laptop screen (33.2 × 21.0 cm), with a viewing distance (no chin rest) of ~60 cm. Stimulus images were 8.0 × 8.0 cm (visual angle ~7.63 × 7.63°). All stimulus images are available at https://doi.org/10.17605/osf.io/ukb5h.

### Experimental procedure

After the neuropsychological and motor assessments, participants individually completed the MR task and the choice-RT task in counterbalanced order. During both tasks, participants sat before a laptop computer, holding a game controller with both hands, thumb side up.

#### Mental rotation task

The MR task consisted of three blocks of each condition (i.e., letters, hands, and feet). The block order was counterbalanced across participants in each age group. Each trial began with a fixation cross for 1.5 s, and then a stimulus was presented. Participants pressed, as accurately and quickly as possible, the game controller’s left button with the left index finger for the normal letter, the left hand, and the left foot, or the right button with the right index finger for the mirrored letter, the right hand, and the right foot.^1^ They were instructed not to move or look at their own hands and feet during the task. (Throughout the MR task, participants’ hands and the game controller were covered by cloth so as not to be visible to the participants. Likewise, participants kept their feet out of sight under the desk.) The next trial began after participants responded or 10 s elapsed. Each condition block consisted of 72 trials (4 stimuli × 6 orientations × 3 repetitions). No feedback was provided during experimental trials. Before each block, participants completed 12 practice trials with feedback. Every practice phase was repeated until participants’ accuracy rate reached 80% (i.e., 10 of 12 trials).

#### Choice reaction time task

The choice-RT task, which served as a control task, was conducted to evaluate motor speed and ability to differentiate two stimuli. Each trial began with a fixation cross for 1.5 s and then Arabic numeral “1” or “2” was presented until participants responded or 10 s elapsed. Participants pressed the left button for “1” or the right button for “2” with the corresponding index finger as accurately and quickly as possible. The experimental task consisted of 72 trials (2 numerals × 36 repetitions) without any feedback. Before experimental trials, participants performed 12 practice trials with feedback.

### Data analysis

#### RT analysis

For the MR task, we excluded incorrect trials (3.69% for the young group and 7.10% for the older group); trials with RT shorter than 200 ms (0.01% for the older group); and trials with correct RT longer than 2,773 ms for the young group (1.65%) or 6,004 ms for the older group (1.81%; the mean plus three *SD*s per group). For the choice-RT task, we excluded incorrect trials (1.04% for the young group and 2.00% for the older group) and trials with correct RT longer than 634 ms for the young group (1.04%) or 937 ms for the older group (1.06%; the mean plus three *SD*s per group). No trial had RT shorter than 200 ms in the choice-RT task. Performance was considered above chance when the overall number of correct responses was above 44/72 trials for each task and condition (one-tailed binomial test, *p* < 0.05). All participants reached this criterion.

Apparent effects of age on raw RTs of the MR task could be confounded by age-related decline in perceptual and motor processing other than MR ability *per se*. Thus, following Zapparoli et al. (2016), we reported results of analysis for ΔRTs, calculated by subtracting each participant’s mean choice RTs from RTs of the MR task. (However, use of raw RTs did not substantially affect overall results of MR data analysis.)

We analyzed ΔRTs using a Bayesian linear mixed-effect model (LMM), which enabled incorporation of both inter-individual and inter-trial variations of data and easy entry of continuous variables (i.e., angle, age, and education), unlike conventional ANOVAs (Lee, 2011; Muto, 2021a). Compared to maximum likelihood (ML) estimation, Bayesian estimation based on Markov Chain Monte Carlo (MCMC) methods is more feasible and tends to provide less biased estimates (McNeish and Stapleton, 2016). A comparable LMM analysis with the ML method showed substantially the same results as reported here, but some models failed to converge well (i.e., estimates were obtained, but error messages appeared). Hence, we decided to apply the Bayesian LMM analysis.

The model contained the following independent variables. As focal within-participant factors, we used angle (0°, 90°, or 135°) and direction of rotation (for letters, counterclockwise = −0.5, clockwise = 0.5; for hands and feet, lateral = −0.5, medial = 0.5). As a focal between-participant factor, we used age group (young = −0.5, older = 0.5) for between-group comparisons (i.e., young vs. older adults) and years of age (continuous variable) for within-group comparisons for older adults. We also entered all possible interactions of these three variables into the model. Education year was included as a covariate. To diminish multicollinearity with interaction terms, we centralized all variables before analyses. We included all possible random effects (i.e., random intercept and slopes) for participants. As prior distributions, we set improper uniform distributions for all parameters.

We conducted a series of Bayesian LMM analyses using R 4.0.3 and the rstan R package (version 2.21.2; Stan Development Team, 2020). The R and Stan scripts are available at https://doi.org/10.17605/osf.io/ukb5h. We obtained MCMC samples from 12 independent chains of 13,000 iterations. For each chain, the initial 500 samples were discarded as warm-ups; then the remaining samples were thinned by retaining every tenth iteration to reduce autocorrelations. Thus, parameters’ posterior distributions were approximated by 15,000 MCMC samples. All Rhat values were below 1.01, confirming the convergence of the parameter estimation.

To evaluate whether each variable had a reliable effect on ΔRT, we used the direction of probability (*pd*) proposed by Makowski et al. (2019) as an index of effect existence. This index is defined as the proportion of the posterior distribution of the same sign as its median’s and varies from 0.50 to 1.00. Unlike Bayes factors or region of practical equivalence (ROPE), the *pd* can be easily calculated without arbitrary heuristics (e.g., choices of desirable prior distributions, practically meaningful ranges). Furthermore, the *pd* can be interpreted very similarly to the *p*-values because they have 1:1 correspondence with each other. For example, two-tailed *p*-values of 0.05, 0.01, and 0.001 correspond to *pd*-values of 0.975, 0.995, and 0.9995, respectively. Thus, consistent with the conventional frequentist framework with *α* = 0.05, we regard a variable as a reliable predictor when its *pd*-value is >0.975. As point and interval estimates, we reported expected *a posteriori* (EAP) estimates and 95% credible intervals (CI) of marginal posterior distributions for interpretation. We also reported marginal and conditional coefficients of determination; i.e., the proportion of variance explained by fixed effects (*R*^2^_m_) and by both fixed and random effects (*R*^2^_c_).

#### Correlation analysis

For correlation analysis, we used Spearman’s rank-order correlation coefficients (*ρ*), which are robust to violations of normality and outliers (unlike Pearson’s correlation coefficients). Since an older participant (87-year-old woman) did not perform the TUG due to difficulty walking, we applied pairwise deletion.

## Results

### Descriptive statistics and their associations with age

**Figure 2:**
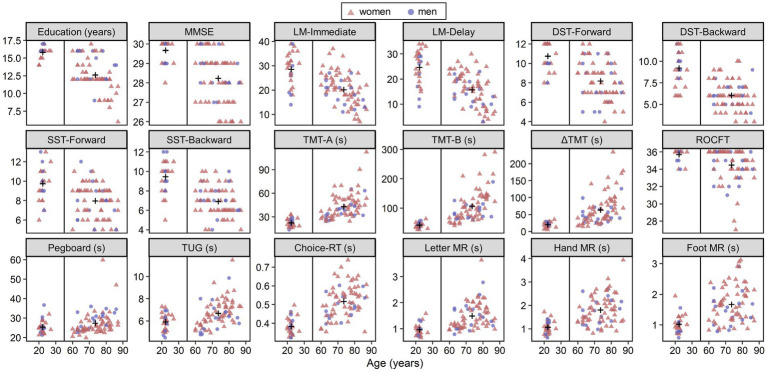
Scatterplots of all indexes as a function of age. The plus signs in panels show group means. LM, logical memory; DST, digit span test; SST, spatial span test; TMT, trail making test; ROCFT, the copy condition of Rey–Osterreich complex figure test; TUG, timed up and go test; RT, reaction time; MR, mental rotation.

Figure 2 shows scatterplots of all indexes (education years, neuropsychological and motor performance, and mean RTs of MR and choice-RT tasks) as a function of age. Plus signs in the panels represent group means. Welch’s *t*-test for group comparisons revealed that young adults showed better mean performance on all tests (absolute values of *d* ranged from 0.37 to 1.83, *p* < 0.046). Within the older group, simple correlation analyses showed age-related decline in performance on all tests (absolute values of *ρ* ranged from 0.253 to 0.640, *p* < 0.046) except for the DST-Backward (*ρ* = −0.232, *p* = 0.052) and the foot MR (*ρ* = 0.213, *p* = 0.074). Education years correlated negatively with age, as shown by both the between-group comparison, *t*(96.4) = −10.18, *d* = −1.60, *p* < 0.001, and the correlation analysis within the older group, *ρ* = −0.520, *p* < 0.001, thus indicating the necessity of treating education years as a covariate to evaluate aging effects.

### Analysis of ΔRT of the MR task

#### Comparison between young and older groups

**Figure 3:**
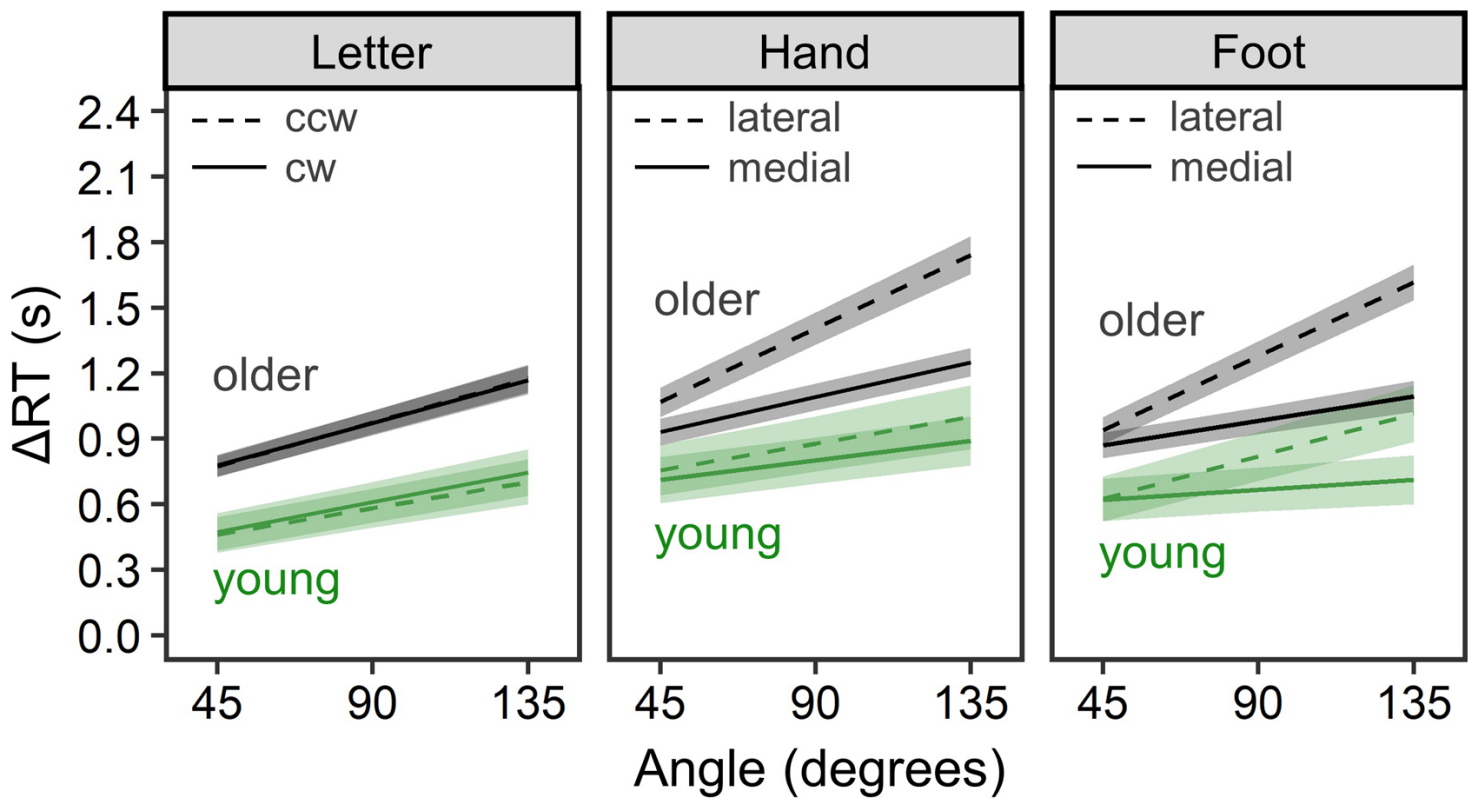
Comparison of mental rotation performance between the young group (*n* = 28) and the older group (*n* = 71). ΔRT is reaction time (RT) of the mental rotation task from which the mean choice RT was subtracted. Lines and error bands represent estimated means and their 95% credible intervals, respectively.

To compare MR performance between young and older groups, we conducted separate Bayesian LMM analyses for ΔRT in each of the letter, hand, and foot conditions. Figure 3 shows estimated mean ΔRT per condition and group, and Table 1 summarizes all parameter estimates.

**Table 1:** Parameter estimates for comparison of the mental rotation ΔRT between the young group (*n* = 28) and older groups (*n* = 71).

Condition	Parameter	EAP	*SD*	95% CI		*pd*	ESS	Rhat
				Lower	Upper			
Letter	Intercept	863.33***	43.05	779.45	949.41	1.000	5,356.5	1.001
	Group (young = −0.5, elderly = 0.5)	377.35***	110.33	163.25	595.59	1.000	6,870.9	1.001
	Direction (ccw = −0.5, cw = 0.5)	3.41	8.16	−12.71	19.51	0.666	7,128.6	1.001
	Angle (degrees)	3.96***	0.25	3.47	4.46	1.000	7,348.9	1.000
	Group × Direction	−14.64	17.98	−49.82	20.83	0.791	11,841.6	1.001
	Group × Angle	1.55**	0.57	0.44	2.67	0.996	9,829.8	1.001
	Direction × Angle	0.00	0.20	−0.39	0.39	0.510	11,117.4	1.000
	Group × Direction × Angle	−0.22	0.43	−1.06	0.64	0.695	11,056.2	1.000
	Education (covariate)	−5.48	17.69	−40.01	29.42	0.624	8,331.4	1.001
Hand	Intercept	1,118.82***	52.08	1,016.22	1,220.95	1.000	7,860.3	1.001
	Group (young = −0.5, elderly = 0.5)	401.16**	138.56	129.42	674.42	0.998	8,163.4	1.001
	Direction (lateral = −0.5, medial = 0.5)	−119.56***	14.33	−147.74	−91.35	1.000	11,092.0	1.000
	Angle (degrees)	4.55***	0.29	3.97	5.13	1.000	11,030.2	1.001
	Group × Direction	−115.68***	31.97	−177.87	−52.67	1.000	11,685.1	1.001
	Group × Angle	3.13***	0.65	1.84	4.41	1.000	12,123.6	1.000
	Direction × Angle	−1.50***	0.26	−2.01	−0.99	1.000	12,760.8	1.000
	Group × Direction × Angle	−1.61**	0.58	−2.74	−0.47	0.997	12,640.3	1.000
	Education (covariate)	−65.82**	23.19	−111.08	−20.44	0.998	9,820.9	1.000
Foot	Intercept	1,008.06 ***	51.00	908.04	1,112.05	1.000	1,919.6	1.006
	Group (young = −0.5, elderly = 0.5)	382.01**	129.49	130.38	644.24	0.998	2,415.2	1.004
	Direction (lateral = −0.5, medial = 0.5)	−123.78***	11.05	−145.85	−102.03	1.000	2,875.1	1.004
	Angle (degrees)	4.27***	0.29	3.70	4.85	1.000	3,747.9	1.004
	Group × Direction	−69.84**	23.63	−116.95	−24.07	0.998	4,208.7	1.002
	Group × Angle	2.32***	0.65	1.06	3.58	1.000	3,768.1	1.002
	Direction × Angle	−2.27***	0.25	−2.77	−1.79	1.000	3,971.1	1.001
	Group × Direction × Angle	−0.87	0.57	−1.97	0.27	0.935	2,760.0	1.004
	Education (covariate)	−41.59^†^	21.41	−83.90	0.53	0.974	5,765.7	1.001

All estimates are in milliseconds. EAP, expected a posterior; SD, standard deviation of posterior distribution; CI, credible interval; pd, probability of direction; ESS, effective MCMC sample size; ***pd > 0.9995 (corresponding to *p* < 0.001), **pd > 0.995 (*p* < 0.01), *pd > 0.975 (*p* < 0.05).

† *pd* > 0.950 (*p* < 0.10).

In the letter condition (*R*^2^_m_ = 0.093, 95% CI = [0.052, 0.146]; *R*^2^_c_ = 0.483, 95% CI = [0.417, 0.553]), overall ΔRTs were longer for the older group than the young group, *b* = 377.35, 95% CI = [163.25, 595.59], *pd* = 1.000. ΔRTs did not reliably differ between clockwise and counterclockwise rotations, *b* = 3.41, 95% CI = [−12.71, 19.51], *pd* = 0.666. In addition, ΔRTs increased with rotational angle of letters, *b* = 3.96, 95% CI = [3.47, 4.46], *pd* = 1.000. This angle effect was larger for the older than for the young group, *b* = 1.55, 95% CI = [0.44, 2.67], *pd* = 0.996. Other interaction terms did not reliably explain ΔRTs (*pd* < 0.792).

In the hand condition (*R*^2^_m_ = 0.195, 95% CI = [0.126, 0.268]; *R*^2^_c_ = 0.509, 95% CI = [0.445, 0.575]), overall ΔRTs were longer for the older group than for the young group, *b* = 401.16, 95% CI = [129.42, 674.42], *pd* = 0.998. Consistent with biomechanical constraints, ΔRTs were longer for lateral than for medial rotation, *b* = −119.56, 95% CI = [−147.74, −91.35], *pd* = 1.000, and for the larger angle, *b* = 4.55, 95% CI = [3.97, 5.13], *pd* = 1.000. A reliable two-way interaction of rotational direction × angle revealed that the angle effect was larger for lateral than for medial rotation, *b* = −1.50, 95% CI = [−2.01, −0.99], *pd* = 1.000. All of these three effects were more salient for older than for young adults, as evidenced by reliable two-way interactions of rotational direction × group, *b* = −115.68, 95% CI = [−177.87, −52.67], *pd* = 1.000; angle × group, *b* = 3.13, 95% CI = [1.84, 4.41], *pd* = 1.000; and reliable three-way interaction of rotational direction × angle × group, *b* = −1.61, 95% CI = [−2.74, −0.47], *pd* = 0.997.

In the foot condition (*R*^2^_m_ = 0.134, 95% CI = [0.086, 0.193]; *R*^2^_c_ = 0.442, 95% CI = [0.381, 0.510]), older adults showed longer overall ΔRTs than young adults, *b* = 382.01, 95% CI = [130.38, 644.24], *pd* = 0.998. Consistent with biomechanical constraints, ΔRTs were longer for lateral than for medial rotation, *b* = −123.78, 95% CI = [−145.85, −102.03], *pd* = 1.000, and for the larger angle, *b* = 4.27, 95% CI = [3.70, 4.85], *pd* = 1.000. The angle effect was larger for lateral than for medial rotation, as revealed by reliable two-way interaction of rotational direction × angle, *b* = −2.27, 95% CI = [−2.77, −1.79], *pd* = 1.000. Compared to young adults, older adults showed a larger medial–lateral effect, as reflected by reliable two-way interaction of rotational direction × group, *b* = −69.84, 95% CI = [−116.95, −24.07], *pd* = 0.998, and a larger angle effect, as reflected by reliable two-way interaction of angle × group, *b* = 2.32, 95% CI = [1.06, 3.58], *pd* = 1.000. We found no reliable three-way interaction of rotational direction × angle × group, *b* = −0.87, 95% CI = [−1.97, 0.27], *pd* = 0.935.

#### Comparison within the older group

**Figure 4:**
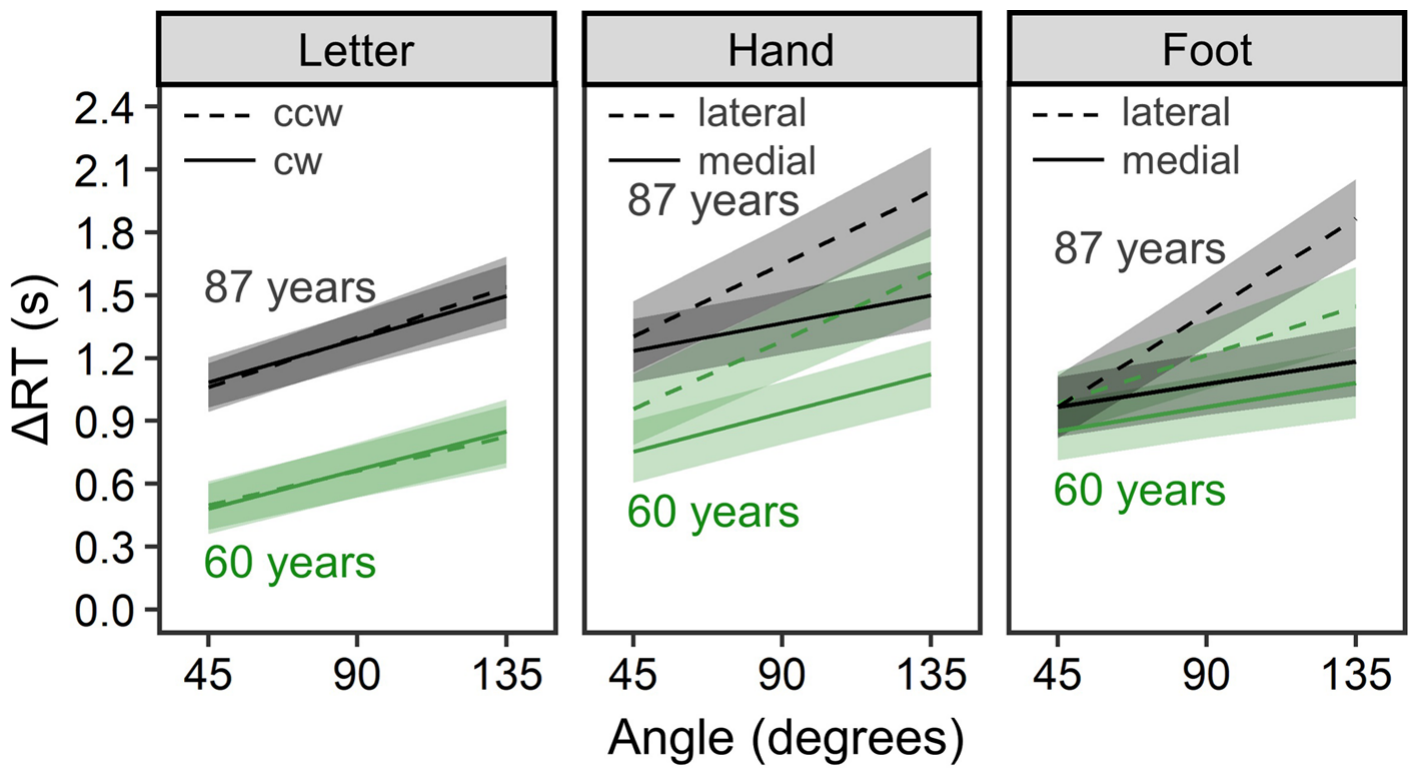
Comparison of mental rotation performance within the older group (*n* = 71). ΔRT is reaction time (RT) of the mental rotation task from which the mean choice RT was subtracted. Lines and error bands represent estimated means and their 95% credible intervals, respectively. Although age was treated as a continuous variable, we substituted the minimal and maximal values of age in our older sample (i.e., 60 and 87 years old) for the estimated models for visualization.

To examine advanced aging effects on MR performance, we extracted the older group’s data and, instead of age group, used models including age in years (a continuous variable) as a fixed effect. Figure 4 shows estimated mean ΔRT per condition and age; for visualization, we substituted minimal and maximal values of age in our older sample (i.e., 60 and 87) for the estimated models (this arbitrary substitution for visualization is irrelevant to analysis results). Table 2 summarizes all parameter estimates.

**Table 2:** Parameter estimates for comparison of the mental rotation ΔRT within older groups (*n* = 71).

Condition	Parameter	EAP	*SD*	95% CI		*pd*	ESS	Rhat
				Lower	Upper			
Letter	Intercept	975.58***	56.12	864.55	1,084.67	1.000	8,110.4	1.001
	Age (years)	23.34**	8.38	7.03	40.02	0.998	9,071.9	1.001
	Direction (ccw = −0.5, cw = 0.5)	−0.88	10.67	−22.08	19.97	0.534	13,676.1	1.000
	Angle (degrees)	4.40***	0.34	3.73	5.06	1.000	8,759.3	1.002
	Age × Direction	−0.23	1.43	−3.02	2.58	0.568	12,864.0	1.000
	Age × Angle	0.04	0.05	−0.05	0.13	0.809	10,115.1	1.001
	Direction × Angle	−0.07	0.26	−0.57	0.45	0.606	13,038.1	1.000
	Age × Direction × Angle	−0.02	0.03	−0.09	0.04	0.735	12,327.0	1.000
	Education (covariate)	27.94	23.46	−17.03	74.13	0.883	10,798.2	1.001
Hand	Intercept	1,292.53***	70.40	1,151.80	1,431.32	1.000	8,503.3	1.001
	Age (years)	14.77	10.73	−5.97	35.60	0.914	10,050.1	1.000
	Direction (ccw = −0.5, cw = 0.5)	−152.89***	19.38	−190.70	−115.53	1.000	11,497.2	1.000
	Angle (degrees)	5.45***	0.40	4.65	6.24	1.000	12,232.7	1.000
	Age × Direction	1.22	2.58	−3.78	6.31	0.684	11,978.8	1.000
	Age × Angle	−0.01	0.05	−0.12	0.09	0.606	13,395.1	1.001
	Direction × Angle	−1.97***	0.36	−2.67	−1.27	1.000	13,575.6	1.000
	Age × Direction × Angle	−0.03	0.05	−0.13	0.06	0.733	14,138.8	1.000
	Education (covariate)	−40.33	30.58	−100.79	18.84	0.907	11,828.0	1.000
Foot	Intercept	1,156.91***	67.46	1,023.84	1,291.13	1.000	6,732.7	1.000
	Age (years)	5.58	10.05	−14.32	25.36	0.715	8,870.8	1.000
	Direction (ccw = −0.5, cw = 0.5)	−143.40***	14.89	−172.97	−114.03	1.000	8,788.1	1.000
	Angle (degrees)	4.94***	0.40	4.16	5.73	1.000	6,311.6	1.001
	Age × Direction	−1.57	1.99	−5.49	2.41	0.785	9,816.7	1.000
	Age × Angle	0.09	0.05	−0.02	0.19	0.945	9,989.7	1.000
	Direction × Angle	−2.53***	0.34	−3.18	−1.87	1.000	10,874.8	1.000
	Age × Direction × Angle	−0.09*	0.05	−0.18	0.00	0.978	13,214.8	1.000
	Education (covariate)	−48.18	29.30	−105.15	9.63	0.949	9,074.0	1.000

All estimates are in milliseconds. EAP, expected a posterior; SD, standard deviation of posterior distribution; CI, credible interval; pd, probability of direction; ESS, effective MCMC sample size. ***pd > 0.9995 (corresponding to *p* < 0.001), **pd > 0.995 (*p* < 0.01), *pd > 0.975 (*p* < 0.05).

In the letter condition (*R*^2^_m_ = 0.073, 95% CI = [0.039, 0.125]; *R*^2^_c_ = 0.460, 95% CI = [0.382, 0.546]), ΔRTs increased with age, *b* = 23.34, 95% CI = [7.03, 40.02], *pd* = 0.998, and with angle, *b* = 4.40, 95% CI = [3.73, 5.06], *pd* = 1.000. ΔRTs were not reliably different between clockwise and counterclockwise rotations, *b* = −0.88, 95% CI = [−22.08, 19.97], *pd* = 0.534. No two- or three-way interactions were detected (*pd* < 0.736).

In the hand condition (*R*^2^_m_ = 0.121, 95% CI = [0.076, 0.181]; *R*^2^_c_ = 0.456, 95% CI = [0.382, 0.538]), we found a reliable medial–lateral effect, *b* = −152.89, 95% CI = [−190.70, −115.53], *pd* = 1.000, and a reliable angle effect, *b* = 5.45, 95% CI = [4.65, 6.24], *pd* = 1.000, as well as a reliable interaction between them, *b* = −1.97, 95% CI = [−2.67, −1.27], *pd* = 1.000—all consistent with biomechanical constraints. However, an age-related increment of mean ΔRT did not reach a reliable level, *b* = 14.77, 95% CI = [−5.97, 35.60], *pd* = 0.914. Age did not reliably interact with rotational direction, *b* = 1.22, 95% CI = [−3.78, 6.31], *pd* = 0.684, or with angle, *b* = −0.01, 95% CI = [−0.12, 0.09], *pd* = 0.606. Additionally, a three-way interaction of rotational direction × angle × age was not reliable, *b* = −0.03, 95% CI = [−0.13, 0.06], *pd* = 0.733.

In the foot condition (*R*^2^_m_ = 0.085, 95% CI = [0.056, 0.127]; *R*^2^_c_ = 0.407, 95% CI = [0.335, 0.488]), biomechanical constraints were evidenced by a reliable medial–lateral effect, *b* = −143.40, 95% CI = [−172.97, −114.03], *pd* = 1.000; a reliable angle effect, *b* = 4.94, 95% CI = [4.16, 5.73], *pd* = 1.000; and their reliable interaction, *b* = −2.53, 95% CI = [−3.18, −1.87], *pd* = 1.000. Overall ΔRTs did not differ reliably across ages, *b* = 5.58, 95% CI = [−14.32, 25.36], *pd* = 0.715, and age did not interact reliably with rotation direction, *b* = −1.57, 95% CI = [−5.49, 2.41], *pd* = 0.785, or angle, *b* = 0.09, 95% CI = [−0.02, 0.19], *pd* = 0.945. However, a three-way interaction of rotational direction × angle × age was reliable, *b* = −0.09, 95% CI = [−0.18, 0.00], *pd* = 0.978, indicating that age-related increase in ΔRTs was larger when a presented foot was biomechanically more awkward (i.e., lateral and large angle rather than medial and small angle).

#### Advanced aging on estimates relative to the young group

**Figure 5:**
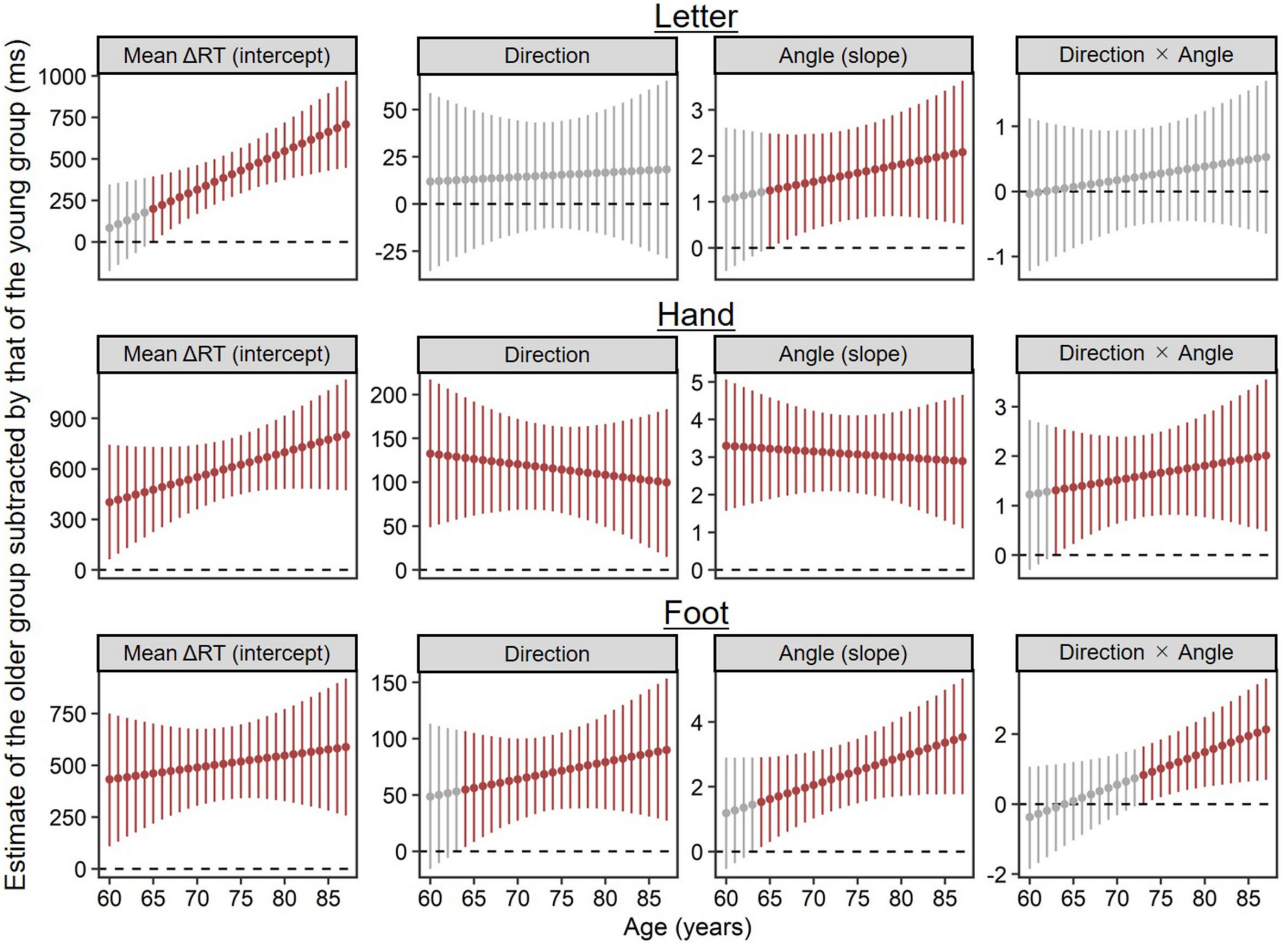
Parameter estimates per age of the older group (*n* = 71) subtracted by corresponding estimates of the young group (*n* = 28), which were calculated using MCMC samples. Higher values indicate stronger contributions for ΔRT relative to the young group. Points and error bars represent estimated means and their 95% credible intervals. Dark red plots show estimates reliably different from the young group’s in the sense that the 95% credible intervals did not include zero.

To supplement the results above for advanced aging effects on ΔRT, we explored at what age parameter estimates (i.e., intercepts, main effects, and interactions) reliably deviated from the young group’s. Specifically, we calculated posterior distributions of parameter estimates per age of the older group by substituting each age (i.e., 60, 61, …, 87) for the previously estimated model and then subtracted from posterior distributions of the young group’s estimates using MCMC samples. Figure 5 shows EAP estimates and 95% CIs of the calculated difference estimates, in which dark red plots indicate estimates reliably different from the young group’s in the sense that the 95% CIs did not include zero.

In the letter condition, both the mean ΔRT (the intercept) and rotation inefficiency (the main effect of angle) were reliably worse at 65 and older than in the young group. The main effect of direction and the two-way interaction of direction × angle did not reliably differ from the young group’s at any age.

In the hand condition, even 60-year-old adults exhibited the longer mean ΔRT (the intercept), the stronger medial–lateral effect (the main effect of direction), and the stronger angle effect (the main effect of angle) than the young group. The two-way interaction of direction × angle was reliably stronger at 63 years and older than in the young group.

Similar to the hand condition, in the foot condition, the mean ΔRT (the intercept) at any age in our older sample (60–87) was reliably longer than in the young group. Both the medial–lateral effect (the main effect of direction) and the angle effect (the main effect of angle) were reliably stronger at 64 years and older than in the young group. Two-way interaction of direction × angle was reliably stronger at 73 years and older than in the young group.

In short, differences from the young group in mean ΔRTs (the intercepts) tended to become reliable at earlier old ages for hand and foot conditions (60 years at the latest) than for the letter condition (65 years). This order is similar to that of angle effects (60 years at the latest for hands; 64 years for feet; 65 years for letters). Within body parts, stimulus orientation effects (the angle effect, the medial–lateral effect, and their interaction) tended to diverge reliably from the young group’s at earlier old ages for hands than for feet.

### Correlation analysis

To examine associations of experimental measures (i.e., choice RT and ΔRT of the MR task) with neuropsychological and motor performance in an exploratory manner, we conducted separate correlation analyses for the young and older groups. For the older group, we calculated Spearman’s partial correlation coefficients, controlling for gender, age, and education years. For the young group, we controlled only for gender.

Table 3 shows partial correlation coefficients for the older group. Better mobility (i.e., shorter time for the TUG) was significantly associated with shorter ΔRTs for letters at all angles (*ρ* > 0.486, *p* < 0.001) and for feet at 45° (*ρ* = 0.267, *p* = 0.026) and at 135° (*ρ* = 0.326, *p* = 0.006), and for choice RT (*ρ* = 0.372, *p* = 0.002). Better performance on the TMT-A was linked to shorter ΔRTs for hands (*ρ* > 0.293, *p* < 0.014) and feet (*ρ* > 0.283, *p* < 0.018) at all angles, but not for letters (*ρ* < 0.186, *p* > 0.121). No other measures correlated significantly with choice RT or ΔRT.

**Table 3:** Spearman’s partial correlation coefficients of experimental measures (choice RT and ΔRT of the MR task) with neuropsychological and motor performance in the older group (*n* = 71; but one did not perform TUG).

Condition		DST		SST		TMT			ROCFT	Pegboard	TUG
		Forward	Backward	Forward	Backward	A	B	Δ			
Choice		−0.096	0.016	0.178	0.117	0.155	0.146	0.068	0.071	0.156	0.372**
Letter MR	45°	−0.042	−0.021	0.090	0.068	0.162	0.088	−0.014	0.134	0.192	0.552***
	90°	−0.068	−0.072	0.106	0.057	0.180	0.042	−0.076	0.101	0.153	0.542***
	135°	−0.060	−0.095	0.061	0.061	0.185	0.121	0.000	0.181	0.143	0.506***
	All	−0.051	−0.065	0.064	0.047	0.150	0.057	−0.050	0.127	0.149	0.486***
Hand MR	45°	−0.097	−0.090	0.107	−0.080	0.381**	0.221^†^	0.064	0.071	0.043	0.174
	90°	−0.103	−0.119	0.127	−0.077	0.396***	0.192	0.032	0.050	−0.039	0.121
	135°	−0.005	−0.144	0.094	−0.084	0.293*	0.228^†^	0.112	0.189	0.023	0.183
	All	−0.062	−0.118	0.121	−0.064	0.357**	0.214^†^	0.068	0.113	−0.004	0.163
Foot MR	45°	0.077	0.066	0.009	0.032	0.303*	0.137	0.006	0.090	0.205^†^	0.267*
	90°	0.017	0.071	0.044	0.024	0.309**	0.133	−0.013	0.081	0.117	0.223^†^
	135°	0.011	0.071	0.051	0.008	0.282*	0.130	0.010	0.111	0.112	0.326**
	All	0.035	0.071	0.052	0.029	0.307**	0.131	−0.006	0.101	0.141	0.275*

Correlation coefficients controlling for gender, age, and education years. “All” means ΔRT averaged across different angles. MR, mental rotation; DST, digit span test; SST, spatial span test; TMT, Trail Making Test; ROCFT, the copy condition of Rey − Osterrieth complex figure test; TUG, Timed Up and Go test. ***p < 0.001; **p < 0.01; *p < 0.05;

† *p* < 0.10.

Table 4 shows partial correlation coefficients for the young group. As for motor performance, greater hand dexterity (i.e., shorter time for the pegboard test) was associated with shorter ΔRTs for letters at 90° (*ρ* = 0.384, *p* = 0.043) and with choice RT (*ρ* = 0.545, *p* = 0.003), while better mobility (i.e., shorter time for the TUG) was associated with shorter ΔRTs for letters at 90° (*ρ* = 0.457, *p* = 0.014) and for hands at all angles (*ρ* > 0.376, *p* < 0.049), and with choice RT (*ρ* = 0.547, *p* = 0.003). As for neuropsychological performance, better performance on DST-Forward was associated with shorter ΔRTs for letters at all angles (*ρ* < −0.478, *p* < 0.010), for hands at 135° (*ρ* = −0.427, *p* = 0.024) and feet at 135° (*ρ* < −0.526, *p* = 0.004), but performance on DST-Backward did not correlate significantly with experimental measures. Better performance on SST-Forward was associated with shorter ΔRTs for hands at 45° (*ρ* = −0.404, *p* = 0.033) and feet at 135° (*ρ* = −0.398, *p* = 0.036), while better performance on SST-Backward was associated with shorter ΔRTs for letters at 135° (*ρ* = −0.408, *p* = 0.031). Time for TMT-A correlated positively with ΔRTs for hands at 45° (*ρ* = 0.438, *p* = 0.020) and at 90° (*ρ* = 0.452, *p* = 0.016), while the TMT-B and ΔTMT had no significant association with experimental measures.

**Table 4:** Spearman’s partial correlation coefficients of experimental measures (choice RT and ΔRT of the MR task) with neuropsychological and motor performance in the young group (*n* = 28).

Condition		DST		SST		TMT			ROCF	Pegboard	TUG
		Forward	Backward	Forward	Backward	A	B	Δ			
Choice		−0.241	−0.101	0.270	−0.114	0.173	0.290	0.269	−0.096	0.545**	0.547**
Letter MR	45°	−0.479**	−0.152	−0.222	−0.288	0.297	0.183	0.093	0.012	0.197	0.283
	90°	−0.510**	−0.127	−0.154	−0.221	0.287	0.197	0.135	−0.069	0.384*	0.457*
	135°	−0.546**	−0.122	−0.106	−0.408*	0.301	0.339^†^	0.257	−0.066	0.330^†^	0.335^†^
	All	−0.538**	−0.123	−0.268	−0.330^†^	0.330^†^	0.243	0.132	−0.044	0.211	0.304
Hand MR	45°	−0.339^†^	0.094	−0.404*	−0.172	0.438*	0.291	0.062	−0.095	0.273	0.376*
	90°	−0.360^†^	0.066	−0.346^†^	−0.141	0.452*	0.266	0.036	−0.119	0.279	0.420*
	135°	−0.427*	0.046	−0.256	−0.047	0.368^†^	0.277	0.141	−0.332^†^	0.256	0.431*
	All	−0.379*	0.053	−0.352^†^	−0.151	0.417*	0.292	0.106	−0.199	0.251	0.430*
Foot MR	45°	−0.315	0.108	−0.331^†^	−0.110	0.065	0.112	0.081	−0.322^†^	0.021	0.216
	90°	−0.313	0.207	−0.139	−0.026	−0.028	−0.018	0.020	−0.242	0.035	0.230
	135°	−0.526**	−0.048	−0.398*	−0.101	0.149	0.145	0.063	−0.296	−0.099	0.271
	All	−0.387*	0.113	−0.282	−0.098	0.081	0.073	0.020	−0.298	−0.010	0.247

Correlation coefficients controlling for gender. “All” means ΔRT averaged across different angles. MR, mental rotation; DST, digit span test; SST, spatial span test; TMT, Trail Making Test; ROCFT, the copy condition of Rey − Osterrieth complex figure test; TUG, Timed Up and Go test. **p < 0.01; *p < 0.05;

† *p* < 0.10.

In both the young and older groups, roughly speaking, high performances on the TMT-A and TUG were associated with short ΔRTs for at least one MR task condition. However, we found somewhat distinct patterns between groups: (1) TMT-A performance was related to ΔRT for hands and feet in the older group, but only for hands in the young group; (2) TUG performance was related to ΔRT for feet and letters in the older group, but for hands and letters (only at 90°) in the young group; and (3) only in the young group, some MR measures (ΔRTs) were associated with memory capacities (DST and SST) and dexterity (pegboard).

## Discussion

This study investigated advanced aging effects on implicit motor imagery evoked by the MR of hands and feet and on visual imagery evoked by the MR of letters. By partial correlation analysis, we also explored how these effects co-occurred with declines in physical motor performance (mobility and dexterity) and in neuropsychological performance. Our findings can be summarized as follows. While the older group consistently showed worse performance for all types of MR and stronger biomechanical constraints for both hand and foot MR than the young group (Figure 3), continuing changes during advanced aging were found only for mean ΔRTs of the letter MR (as the main effect of age) and for strength of biomechanical constraints in the foot MR (as the three-way interaction of rotational age × direction × angle; see Figure 4). Further analysis suggested that the performance difference from young adults emerged at relatively earlier ages for the hand and foot MR than for the letter MR (Figure 5). In partial correlation analysis, we observed both similar and distinct patterns between age groups for associations of MR performance with motor (TUG and pegboard task) and neuropsychological (especially the TMT-A and DST-Forward) performance. We discuss these findings in detail below.

### Differences between young and older adults

Between-group comparisons confirmed age-related declines in both visual and implicit motor imagery (Figure 3). In the letter MR related to visual imagery, older adults showed a stronger angle effect and longer mean ΔRTs than young adults. This replicates previous studies on declines in efficiency of visual or object-based transformations (Cerella et al., 1981; Berg et al., 1982; Dror and Kosslyn, 1994; Gondo et al., 1998; Briggs et al., 1999; Band and Kok, 2000; De Simone et al., 2013; Iachini et al., 2019; Zhao et al., 2019; Muto et al., 2020).

In the hand MR, both groups showed angle and medial–lateral effects reflecting biomechanical constraints (Sekiyama, 1982; Parsons, 1987), thus implying use of motor imagery. Our older participants aged 74 on average showed longer ΔRTs than young participants, and importantly, the group difference was pronounced when hands were presented in biomechanically awkward postures. This result closely resembles that of Saimpont et al. (2009), who sampled older adults aged 78 on average and analyzed raw RTs, rather than of Zapparoli et al. (2016), who sampled younger older adults aged 61 on average and failed to detect any group difference in ΔRTs (i.e., RTs of MR minus choice RTs). Thus, our finding suggests that age-related declines in hand MR performance do not manifest easily at earlier older ages, and the aging effect could be attributed not to general motor and perceptual slowing (i.e., long choice RTs) but to hand motor imagery deficits (i.e., longer mean ΔRT and pronounced biomechanical constraints).

Using the foot MR, we first investigated the aging process of implicit motor imagery for the lower limbs. The foot MR task yielded qualitatively the same patterns as the hand MR, with the exception only of lacking reliable three-way interactions of direction × angle × group. Older adults showed longer mean ΔRTs and stronger effects of biomechanical constraints than young adults, confirming age-related decline in implicit foot motor imagery. Thus, the present study extended existing knowledge on age-related deficits in implicit motor imagery by demonstrating that they are not limited to the hands and but emerge also for the feet or lower limbs.

In short, we clearly confirmed age-related deficits in implicit motor imagery as well as in visual imagery. Enhanced susceptibility to biomechanical constraints raises a possibility that, as already used for patient rehabilitation (Dijkerman et al., 2004; Grangeon et al., 2012), the MR of hands and feet can help assess and train older adults’ physical motor skills.

### Advanced aging effects on motor and visual imagery

Our analyses of adults aged 60–87 years examined changes in visual and implicit motor imagery during advanced aging (Figure 4). For visual imagery, we found an advanced age-related increase in mean ΔRTs of the letter MR, but no reliable advanced aging effect on ΔRTs as a function of angle. The lack of difference in the angle effect aligned with Gondo et al. (1998), who reported no difference in RT slopes for the letter MR between young–old (64–74 years) and old–old (75–92 years) groups. In contrast, studies using paper-and-pencil MR tests with three-dimensional cubes reported decreased MR performance during advanced aging (Borella et al., 2014; Muto et al., 2020). This inconsistency might arise from methodological differences in task demand, stimulus type, and/or dependent variable. Because we controlled for general motor and perceptual declines using choice RTs, slowing of the mean ΔRTs may reflect reduced resources, for instance, switching costs for rotation and non-rotation strategies between trials (Ilan and Miller, 1994; Muto, 2021b). Thus, our letter MR results should not be interpreted as evidencing advanced aging declines in visual transformations *per se*.

For advanced aging in implicit motor imagery, we found only the strengthened synergistic effect of the angle and the rotational direction (i.e., medial or lateral) in the foot MR. This suggests that susceptibility to the lower limbs’ biomechanical constraints continues to increase during late adulthood. In contrast, we found no reliable advanced aging effect for the hand MR. Contrast between hands and feet also appeared in our partial correlation analysis, showing that older adults’ time on the TUG correlated positively with ΔRT of the foot (and letter) MR, but not of the hand MR. We propose two possible accounts for the difference between hands and feet (not mutually exclusive). First, older adults could adopt a visual strategy for the hand MR to compensate for impaired motor imagery (Zapparoli et al., 2016), but that strategy is likely less effective for the foot MR because of seeing the feet less often than the hands, thus leading to increased reliance on motor imagery for the foot MR. Second, the foot MR could be more sensitive to the lower limb impairments often experienced by older adults than the hand MR, because the hand and foot MR reflect the corresponding body image (Ionta et al., 2007). To test these accounts and elucidate detailed mechanisms, future aging studies should take advantage of the foot MR, in addition to the hand MR, as the present study did.

We also explored at what age older adults’ MR performance reliably deviated from that of young adults (Figure 5). In our sample, implicit motor imagery began declining at earlier old ages than visual imagery. This is consistent with previous findings that differences between young and older groups were less detectable for object-based than egocentric transformations (De Simone et al., 2013; Kaltner and Jansen, 2016), suggesting differing aging trajectories for visual and motor imagery. This is also consistent with the view of visual dominance or older adults’ tendency to rely more heavily on visual processing than on other modalities (Costello and Bloesch, 2017), in that relatively intact visual imagery could compensate for motor imagery deficits (Zapparoli et al., 2016).

Within body parts, the group difference in biomechanical constraint effects tended to emerge at earlier old ages for hands than for feet (Figure 5). Together with the result that the advanced aging effect on biomechanical constraints was found only for the foot MR (Figure 4), possibly, declines in implicit motor imagery for the lower limbs begin to emerge at relatively late old ages but then progress rapidly. Due to model and data restrictions (i.e., parameter identification problems), such a nonlinear trajectory could not be tested in the present study, but it is worthy of future research.

Which neural substrates are involved in the aging process of three MR types? The MR of body parts activates not only brain areas responsible for motor planning and execution, such as the sensorimotor and premotor cortices, but also areas relevant to visuospatial processing, such as the parietal cortex and the intraparietal sulcus (Parsons et al., 1995; Kosslyn et al., 1998; Sekiyama et al., 2000; Vingerhoets et al., 2002; de Lange et al., 2006; Osuagwu and Vuckovic, 2014), which are also crucial for mental visual transformations (for a review, see Zacks, 2008). This fact suggests that while parietal regions’ dysfunction worsens MR performance regardless of stimulus type, motor-related regions’ dysfunction further impairs the hand and foot MR. A post-hoc correlation analysis indirectly supported this notion: When controlling for gender, age, and education years, older adults’ mean ΔRTs strongly correlated between the hand and foot MR (*ρ* = 0.788, *p* < 0.001) and moderately correlated between the letter and hand MR (*ρ* = 0.510, *p* < 0.001) and between the letter and foot MR (*ρ* = 0.571, *p* < 0.001), consistent with common and distinct neural bases of declines in visual and implicit motor imagery. These correlations cannot be accounted for merely by neural dedifferentiation (Goh, 2011) because no neuropsychological measures correlated with ΔRTs of both visual and motor imagery conditions. Further neuroimaging studies should corroborate these considerations.

Notably, apparent preservation of MR performance during late adulthood does not necessarily indicate absence of advanced aging effects because the same observation could be made when deficits in motor imagery *per se* (increasing biomechanical effects) are canceled by enhanced visual compensation strategies without motor imagery (decreasing biomechanical effects). Since the present study was cross-sectional, we should also consider that selection bias could mitigate advanced aging effects; our older participants were limited to those who survived to their age and were healthy enough to participate in the study. In addressing these issues, developing behavioral and neuroscientific procedures that can disentangle visual and motor processing and minimize potential bias are important.

### Associations between imagery and sensorimotor performance

We conducted correlation analysis to explore whether older adults have co-occurring deficits in imagery and motor performance. Although simple correlation analysis confirmed age-related declines in almost all measures (Figure 2), a limited number of indices had significant partial correlations with older adults’ MR performance when controlling for gender, age, and education years (Table 3). This indicates that apparent co-occurring deficits do not imply the existence of shared mechanisms.

Among neuropsychological measures, only TMT-A performance was positively associated with older adults’ performance for the hand and foot MR but not for the letter MR. TMT-A performance is considered to reflect low-level perceptual and motor processing, such as visual scanning and motor speed, rather than executive function involved in the TMT-B (Corrigan and Hinkeldey, 1987; Drane et al., 2002), memory components involved in the DST and SST, and visuoconstructional processes involved in the ROCF. This result is at least consistent with the embodied cognition framework, which posits that high-level cognitive functions (e.g., imagery) are tightly linked to primitive sensorimotor systems (Wilson, 2002; Waller, 2014). Selective associations between the TMT-A and body MR performance perhaps reflects stronger reliance on motor processes for the hand and foot MR than for the letter MR; this raises the possibility that motor-related brain areas (e.g., premotor, supplementary motor, cerebellar regions; see Parsons et al., 1995; Kosslyn et al., 1998; Sekiyama et al., 2000; Vingerhoets et al., 2002; de Lange et al., 2006; Osuagwu and Vuckovic, 2014) are responsible for co-occurring deficits in TMT-A performance and implicit motor imagery.

Mobility measured by the TUG had associations with ΔRT of the letter MR (for all angles) and the foot MR (except for 90°) as well as with choice RT, while dexterity measured by the pegboard task had no reliable associations with any RT measures. These results are partially consistent with Kawagoe and Sekiyama (2014), who reported that older adults’ TUG performance correlated with *N*-back task performance for visually encoded stimuli (i.e., faces and locations) when controlling for age and general cognitive function, while pegboard test performance had no such correlations. In a subsequent neuroimaging study, Kawagoe et al. (2015) found that older adults with lower TUG performance showed more activities in prefrontal regions and fewer activities in subcortical structures, including the putamen, thalamus, and cerebellum, during the 1-back task; this suggested a prefrontal compensation mechanism for subcortical deficits in older adults with low mobility. Analogically, we could assume that mobility reduction co-occurs with brain alterations associated with impaired MR and delayed choice RT, which then motivate use of an alternative compensatory strategy. Because of the exploratory nature of the analysis, this account remains speculative and the question remains as to why only the hand MR lack a correlation with TUG performance. Nonetheless, the present study first highlighted mobility’s potential link to older adults’ motor and visual imagery.

These results appear to contradict the findings of Jansen and Kaltner (2014), who reported no significant associations of TUG performance with RTs for object-based MR of letters (same/different judgment) or for egocentric MR of whole bodies (left–right identification). Rather, they found association between RT for object-based whole body MR (same/different judgment) and balance. A possible reason for lack of correlation between the TUG and object-based MR performance in their study might be that their participants were younger (60–71 years) than our older participants (60–87 years), so the aging process had not sufficiently progressed. This is consistent with the notion that deficits in object-based transformations emerge at later old ages than in egocentric transformations (De Simone et al., 2013; Kaltner and Jansen, 2016). For egocentric MR, distinguishing between effector-based and perspective-based transformations is important (for a review, see Zacks and Michelon, 2005). Whereas the MR of body parts elicits transformations of corresponding body images (effector-based), left–right identification of whole bodies induces transformations of whole bodies (perspective-based) rather than those of specific body parts. Given distinct embodied processes between them (Muto et al., 2018), it is not surprising that TUG performance correlates with implicit foot motor imagery as in the present study but not with whole-body transformations as in the previous study. This consideration clarifies the need to identify which body function is selectively or uniformly linked to different embodied processes.

Comparisons with young adults may be helpful in understanding older adults’ observed associations between sensorimotor and MR performance. Similar to older participants, young participants’ TUG performance correlated with choice RT and MR performance. However, in contrast with older participants, associations were found for hands (all angles) and letters (only 90°) but not for feet. In addition, performance on the pegboard task was associated with RT measures (specifically, choice RT and ΔRT for letters at 90°) for young participants only. In neuropsychological measures, TMT-A performance correlated with ΔRT for hands but not for feet (in older participants, both correlated with TMT-A performance). Moreover, only young participants showed significant associations between some MR measures and memory capacities (DST and SST). These differences might be explained by relatively small individual differences in young adults who had not yet been affected by aging, making their performance susceptible to confounding factors like motivation and fatigue rather than motor functions. The partial correlation coefficient between completion times for the TUG and pegboard tests was consistently higher for the young group (*ρ* = 0.595, *p* < 0.001, controlling for gender) than for the older group (*ρ* = 0.265, *p* = 0.027, controlling for gender, age, and education years), which suggests underlying common factors for young adults. Additionally, we should consider the possibility that group differences were at least partially due to differing statistical power (i.e., smaller sample sizes for the young than for older groups). Nonetheless, pursuing the possibility of distinct embodied processes between young and older adults might be fruitful.

In conclusion, we provided some evidence for co-occurring age-related deficits in implicit motor imagery and physical motor functioning during advanced aging, as predicted by the embodied cognition framework. We further indicated distinct aging trajectories of visual imagery, hand motor imagery, and foot motor imagery. Although this study was somewhat exploratory, the candidate neural mechanisms that we proposed would help guide future research for elucidating interdependence between cognitive and motor systems; this might also lead to clinical applications, for instance, in rehabilitation. Together with findings on child development of motor imagery (Sekiyama et al., 2014; Iachini et al., 2019), these findings on advanced aging should contribute to a comprehensive model for lifespan development of embodied processes.

## Data availability statement

The datasets presented in this study can be found in online repositories. The names of the repository/repositories and accession number(s) can be found at: https://doi.org/10.17605/osf.io/ukb5h.

## Ethics statement

The studies involving human participants were reviewed and approved by the Ethical Committee of Kumamoto University. The patients/participants provided their written informed consent to participate in this study.

## Author contributions

HM contributed to conceptualization, data curation, formal analysis, methodology, visualization, writing—original draft, and writing—review and editing. MS contributed to conceptualization, data curation, investigation, methodology, resources, and writing—review and editing. KS contributed to conceptualization, funding acquisition, methodology, project administration, resources, supervision, and writing—review and editing. All authors contributed to the article and approved the submitted version.

## Funding

This work was supported by JSPS Grants-in-Aid for Scientific Research (A) (nos. 25245068 and 21H04422) to KS and a JSPS Grant-in-Aid for Scientific Research (S) (no. 16H06325) to KS.

## Conflict of interest

The authors declare that the research was conducted in the absence of any commercial or financial relationships that could be construed as a potential conflict of interest.

## Publisher’s note

All claims expressed in this article are solely those of the authors and do not necessarily represent those of their affiliated organizations, or those of the publisher, the editors and the reviewers. Any product that may be evaluated in this article, or claim that may be made by its manufacturer, is not guaranteed or endorsed by the publisher.

## Footnotes

^1^During the instruction phase, the experimenter explained the response buttons in the order of the left and right hands regardless of task type. To avoid confusion, we assigned what seemed to be "default" responses (i.e., "1" for the choice-RT task and "normal" for the letter MR) for the left hand.
